# Robust and Efficient Mediation Analysis via Huber Loss

**DOI:** 10.1017/psy.2024.28

**Published:** 2025-01-13

**Authors:** WenWu Wang, Xiujin Peng, Tiejun Tong

**Affiliations:** 1 School of Statistics and Data Science, Qufu Normal University, Qufu, China; 2 Department of Mathematics, Hong Kong Baptist University, Kowloon Tong, Hong Kong

**Keywords:** data-driven tuning constant, Huber loss, indirect effect, iteratively reweighted least-squares, M-regression

## Abstract

Mediation analysis is one of the most popularly used methods in social sciences and related areas. To estimate the indirect effect, the least-squares regression is routinely applied, which is also the most efficient when the errors are normally distributed. In practice, however, real data sets are often non-normally distributed, either heavy-tailed or skewed, so that the least-squares estimators may behave very badly. To overcome this problem, we propose a robust M-estimation for the indirect effect via a general loss function, with a main focus on the Huber loss which is more slowly varying at large values than the squared loss. We further propose a data-driven procedure to select the optimal tuning constant by minimizing the asymptotic variance of the Huber estimator, which is more robust than the least-squares estimator facing outliers and non-normal data, and more efficient than the least-absolute-deviation estimator. Simulation studies compare the finite sample performance of the Huber loss with the existing competitors in terms of the mean square error, the type I error rate, and the statistical power. Finally, the usefulness of the proposed method is also illustrated using two real data examples.

## 1 Introduction

In social sciences and related areas, the effect of an exposure on the outcome variable is often mediated by an intermediate variable. Mediation analysis aims to identify the direct effect of the predictor on the outcome and the indirect effect between the same predictor and the outcome via the change in a mediator (MacKinnon, 2008). Since the seminal paper of Baron and Kenny (1986), mediation analysis has become one of the most popular statistical methods in social sciences. Empirical applications of mediation analysis have dramatically expanded in sociology, psychology, epidemiology, and medicine (Lockhart et al., 2011; Newland et al., 2013; Ogden et al., 2010; Richiardi et al., 2013; Rucker et al., 2011). In practice, however, researchers have found that the assumptions of traditional mediation analysis methods, e.g., normality and no outliers, do not match the data they collected, which may lead to misleading results (Preacher, 2015; Yuan & MacKinnon, 2014). To overcome the problem, it is often required to adopt some sophisticated models for mediation analysis (Frölich & Huber, 2017; Lachowicz et al., 2018; VanderWeele & Tchetgen, 2017). For more details on mediation analysis, one may refer to the recent books including, for example, MacKinnon (2008), VanderWeele (2015), and Hayes (2023).

One important issue in mediation analysis is to conduct the inference on the indirect effect, with a main focus on testing its statistical significance. In this direction, the first approach is the causal steps approach (Baron & Kenny, 1986), which specifies a series of tests of links in a causal chain. Moreover, some variants of this method that test three different hypotheses have also been proposed (Allison, 1995; Kenny et al., 1998). The second approach is the difference in coefficients approach (Freedman & Schatzkin, 1992), which takes the difference between a regression coefficient before and after being adjusted by the intervening variable. The third approach is the product of coefficients approach which involves paths in a path model (MacKinnon et al., 1998, 2004; Sobel, 1982). MacKinnon et al. (2002) compared 14 methods of testing the statistical significance of the indirect effect and found that the difference in coefficients approach and the product of coefficients approach have a better control on the type I error rate as well as a higher power in most cases. And between them, the product of coefficients method is more widely used mainly thanks to its clear causal path explanation (MacKinnon et al., 2004; Preacher & Hayes, 2008; Preacher & Selig, 2012; Yuan & MacKinnon, 2014).

To estimate the indirect effect, the least-squares (LS) regression is routinely applied, which is also the most efficient when the errors are normally distributed. In practice, however, real data sets are often non-normally distributed, either heavy-tailed or skewed (Field & Wilcox, 2017). As an example, Micceri (1989) examined 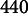 data sets from the psychological and educational literature and found that none of them were normally distributed at the 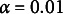 significance level. When applied to non-normal data sets, the LS estimators may behave very badly (Huber & Ronchetti, 2009). To circumvent such drawbacks, some robust approaches have recently emerged in the mediation literature. Zu and Yuan (2010) adopted the local influence function to identify the strongly-affected outliers. Yuan and MacKinnon (2014) proposed the least-absolute-deviation (LAD) regression when the errors are heavy-tailed, and moreover, Wang and Yu (2023) established the statistical theory for the LAD estimation of the indirect effect. Lastly, as claimed by Preacher (2015), mediation analysis for non-normal variables has become an active research field.

To move forward, it is noteworthy that the LS and LAD estimators are special cases of the M-estimators, which minimize a specified loss function (Hansen, 2022; Serfling, 2001). Another popular loss function in the M-regression is known as the Huber loss function, which utilizes a tuning parameter to adjust the tail of the standard normal distribution (Huber, 1964). This tuning parameter controls the trade-off between the efficiency and robustness. Wang et al. (2007) found that the Huber loss function with the optimal tuning parameter can greatly improve the efficiency when maintaining the robustness. To the best of our knowledge, little work has been done on estimating the indirect effect from the perspective of the optimal loss.

This article proposes to further advance the literature by developing robust estimation of the indirect effect. To be specific, our approach mainly alleviates effects in the response variable and implicitly assumes that there is no large leverage points in the independent variables. In Section 2, we introduce the M-regression in the simple mediation model with a general loss function. An iteratively reweighted least-squares algorithm is also proposed to numerically solve the M-regression, as well as to construct two robust confidence intervals. In Section 3, we propose a data-driven approach to select the optimal tuning constant, and moreover study the statistical properties specifically for the Huber loss. In Section 4, we conduct simulation studies to assess the finite sample performance of the Huber loss and compared it with the existing competitors used in mediation analysis. We further illustrate the advantages of our method by an empirical example in Section 5, and conclude the article in Section 6 with some discussion.

## 2 Simple mediation model

**Figure 1:**
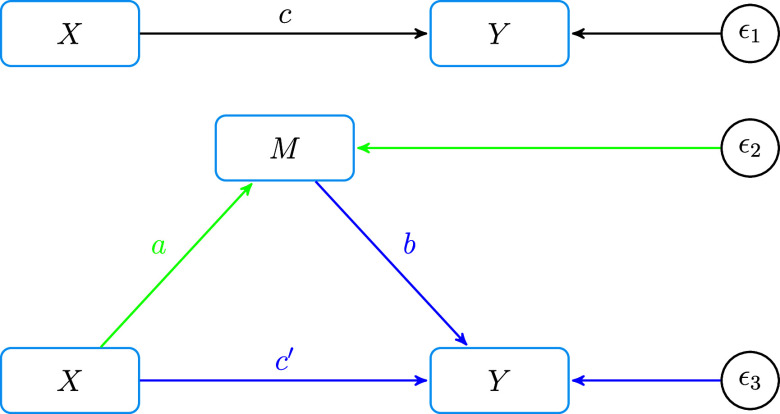
Causal diagram of the simple mediation model.

The simplest mediation model is given in Figure 1, where *X* is the independent variable, *Y* is the dependent variable, and *M* is the mediating variable that mediates the effects of *X* on 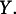 Given the observations 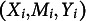 for 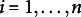 , this simple mediation model consists of three linear regression equations as 
(1)

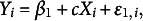



(2)

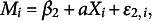



(3)

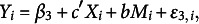

where *c* represents the total effect of *X* on *Y*, *a* represents the relation between *X* and *M*, 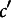 represents the direct effect of 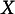 on *Y* after adjusting the effect of *M*, *b* represents the relation between *M* and *Y* after adjusting the effect of *X*, and the random errors 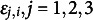 , are independent of the corresponding regressors.

### 2.1 M-regression

To alleviate the effects of influential observations in the least-squares fitting, we adopt the M-regression to estimate the regression parameters, which can be regarded as a generalization of the maximum likelihood estimation as follows: 
(4)

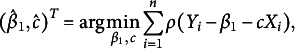



(5)

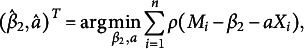



(6)

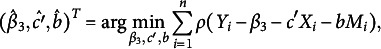

where 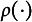 is the loss function with three properties: (i) non-negativity such that 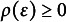 with 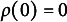 , (ii) symmetricity such that 
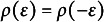
 , and (iii) monotonicity such that 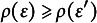 for any 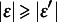 .

Let 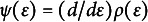 be the first derivative of the loss function, referred to as the influence curve. Let also 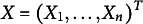 , 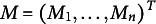 , 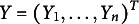 , 
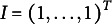
 , 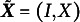 , and 
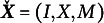
 . For large samples, we further assume that 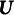 is the limiting matrix of 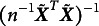 , and 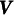 is the limiting matrix of 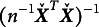 . Then by Huber and Ronchetti (2009), we have the following asymptotic normality for the M-estimators of the regression parameters.

Lemma 1. For the mediation model linked with (1)–(3), under the regularity conditions given on pages 163–164 of Huber and Ronchetti (2009), the M-estimators in (4)–(6) are all normally distributed: 

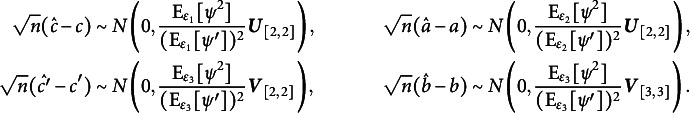

Finally, based on the M-estimators in (4)–(6), we can define two new estimators of the indirect effect: one is the difference estimator 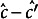 and the other is the product estimator 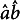 .

### 2.2 Solution to M-regression

For a general loss 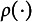 , noting that the M-estimator may not have an explicit expression, a numerical solution is often required. To present our algorithm, we will focus only on (4) since the same algorithm can be extended to solve (5) and (6) as well. Differentiating the objective function 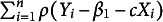 with respect to 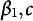 and setting the partial derivatives to be zero, it yields a system of two estimating equations as 

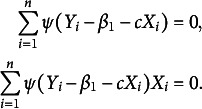

Further by introducing the weight function 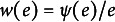 , the estimating equations can be rewritten as 

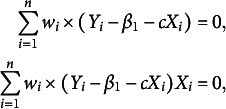

where 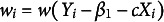 . Solving these two equations is equivalent to minimizing 

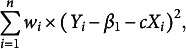

which is a weighted LS problem. Moreover, an iteratively reweighted least-squares (IRLS) algorithm can be appropriate to obtain the numerical solution of the regression coefficients, because the weights depend on the regression coefficients, and the regression coefficients in turn depend on the weights (Holland & Welsch, 1977). To also handle the multiple-minima problem, in case it has, we choose several different points in the parameter space as the initial estimates, in such a way to get a higher confidence to obtain the true global minimum (Green, 1984). More specifically, the IRLS algorithm for our problem is as follows.

**Figure:**
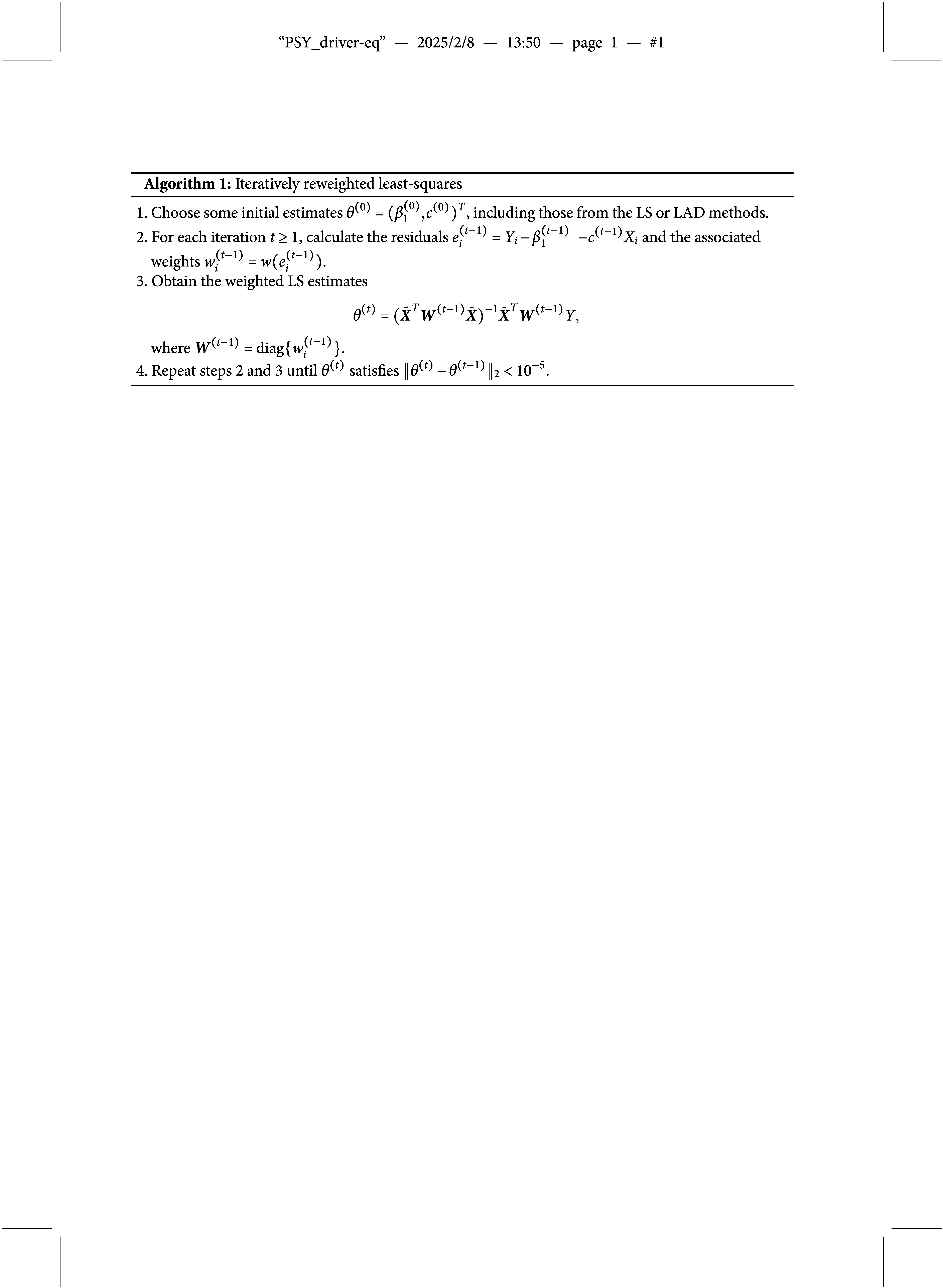

### 2.3 Error conditions for model consistency

When is the product of parameters 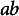 equal to the difference in parameters 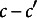 in population? This is an important question in mediation analysis since it uncovers the relationship between the indirect, direct and total effects (Wang et al., 2023; Wang & Yu, 2023; Yuan & MacKinnon, 2014).

Note that the three regression equations, (1)-(3), are interrelated in the simple mediation model. By substituting (2) into (3), we have 
(7)

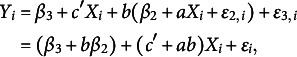

where 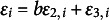 . Assume that 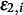 and 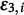 are independent and symmetrically distributed with median 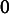 , then 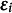 is also symmetric with 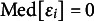 (see Proposition 1 in Wang and Yu (2023)). In addition, let 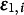 also be symmetrically distributed with 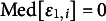 . Then by (1) and (7), 

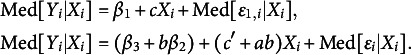

Noting also that the random errors are independent of the corresponding regressors as assumed in Section 2.1, we have 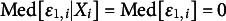 and 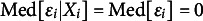 , and moreover, 

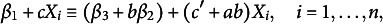

which further yields that 
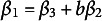
 and 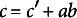 . Finally, by comparing (1) and (7), we also have 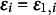 . For convenience, we summarize the above result in Theorem 1.

Theorem 1. In the simple mediation model, given the independence of the errors and the corresponding regressors, we further assume that the errors are independent and symmetrically distributed with a unique median 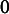 for 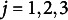 . Then we have 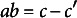 , which builds an equality between the indirect effect, direct effect and total effect.

Remark 1. Many error distributions satisfy the error assumption in Theorem 1. For instance, when 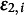 and 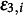 are independent and normally distributed, Yuan and MacKinnon (2014) discussed the model consistency. Wang and Yu (2023) further discussed the consistency conditions for the LAD loss and obtained the similar equality as in Theorem 1.

### 2.4 Inference based on confidence interval

There are two estimators for the indirect effect: 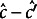 and 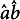 . Unlike the equivalence of the two LS estimators (MacKinnon et al., 1995; Wang et al., 2023), the two M-estimators of the indirect effect for a general loss are not the same in general, that is, 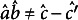 . Simulation studies show that the product estimator is often more efficient than the difference estimator (see Appendix A0). Interestingly, the same conclusion can also be seen when the LAD loss is applied (Wang & Yu, 2023). In view of this, we thus consider the null hypothesis 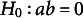 . To test whether 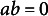 , there are two common methods in the literature including the parameter method (Sobel, 1982) and the nonparametric resampling method (MacKinnon et al., 2004; Preacher & Selig, 2012).

To move forward, our first method is to perform a robust Sobel test. Given the robust estimates 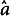 and 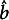 , we define the robust test statistic as 

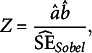

where 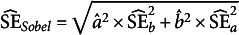 , and 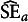 and 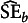 are the standard errors (SEs) of 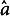 and 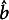 , respectively. Following Theorem 1, the two SEs can be estimated by 

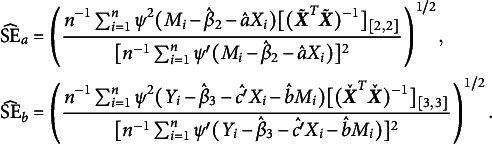

Moreover, the normal-based 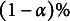 CI of 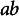 can be constructed as 

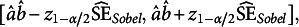

where 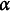 is the significance level, and 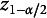 represents the 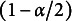 quantile of the standard normal distribution. Note however that, when *a* and *b* are small, the sampling distribution of 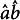 may not be normal (MacKinnon et al., 2004; Wang et al., 2023). Thus to obtain an accurate CI, critical values of the distribution of 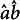 can be obtained by Monte Carlo simulation study (Meeker et al., 1981; Meeker & Escobar, 1994). In fact, one can easily obtain these critical values via inputting 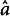 , 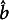 , 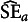 and 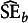 into an R procedure medci() which was introduced by Tofighi and MacKinnon (2011).

Our second method to construct CI is the bootstrap method based on resampling. The bootstrap method is nonparametric and robust in the sense that it does not need to estimate the SEs. First, we repeatedly resample the original dataset with replacement (Efron & Tibshirani, 1993); second, we estimate the indirect effect for each bootstrap sample using our proposed Huber method; third, we construct the CI by the percentile bootstrap (PRCT) as 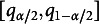 , where 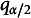 is the 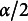 quantile of the empirical distribution of the indirect effect. To adjust and remove the potential estimation bias, the bias-corrected and accelerated bootstrap (BCa) is an important variation (Efron, 1987; Efron & Tibshirani, 1993). In general, the BCa method can yield a more accurate CI than the PRCT method when the true parameter value is not the median of the distribution of the bootstrap estimates (MacKinnon et al., 2004).

## 3 Robust and efficient estimation via Huber loss

From a likelihood perspective, the best loss function would be the negative log-likelihood function (Schrader & Hettmansperger, 1980). Nevertheless, since the likelihood function is often unknown, one needs to specify an appropriate loss function in real applications. In this section, we study the robust and efficient estimation using the Huber loss with the optimal choice of tuning parameter. Note that our methodology is general and can also be extended to other loss functions.

### 3.1 Huber Loss

The Huber loss, as defined in Huber (1964), is given as 

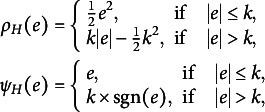

where 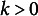 is the tuning parameter. A smaller value of *k* produces more resistance to outliers, but at the expense of lower efficiency when the error is normal. For instance, by letting 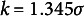 with 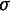 being the standard deviation of the error, it will yield a 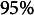 efficiency for the normal errors, which is also resistant to outliers with a breakdown point of 5.8%. Moreover, the standard deviation 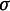 can be estimated robustly by the median absolute deviation (MAD) as 

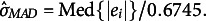



For any error 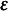 , we denote 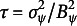 as the asymptotic variance of the Huber estimator (Huber, 1964), where 
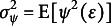
 and 
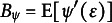
 . We then minimize the 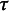 value to determine the optimal 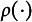 . For the Huber loss with a given *k*, we have 

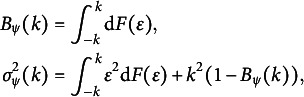

where 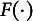 is the cumulative distribution function of 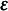 .

Remark 2. As 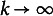 , the Huber loss becomes the LS loss so that 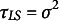 , where 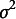 is the variance of the error distribution. As 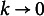 , the Huber loss becomes the LAD loss so that 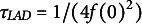 , where 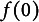 is the density value of the error distribution at 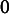 . Based on the observational data, the optimal tuning constant can be selected to obtain the smallest estimation variance. From this viewpoint, the Huber estimator is more efficient than its competitors when dealing with the unknown and complex error distributions.

### 3.2 Optimal Tuning constant

As is known, the tuning parameter *k* of the Huber loss can have a great impact on the estimation efficiency. When the error is normally distributed without contamination, the best choice of *k* is 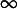 . On the other hand, when the error follows a heavy-tailed distribution such as the *t* distribution, then *k* tends to be a small value close to 0.

We adopt a numerical method proposed by Wang et al. (2007) to select the optimal tuning constant, which minimizes the asymptotic variance of the estimator. For the Huber loss, the optimal *k* minimizes the efficiency factor 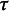 with a three-step procedure as follows. First, we compute 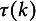 for a range of *k* values, i.e., 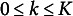 by 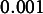 , where *K* is a positive number, e.g., 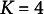 . Second, we select the optimal *k* as 

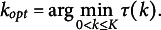

Lastly, we compute the minimum value 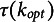 . In Appendix B, we provide an R procedure to obtain the optimal tuning constant with a known error distribution.

**Figure 2:**
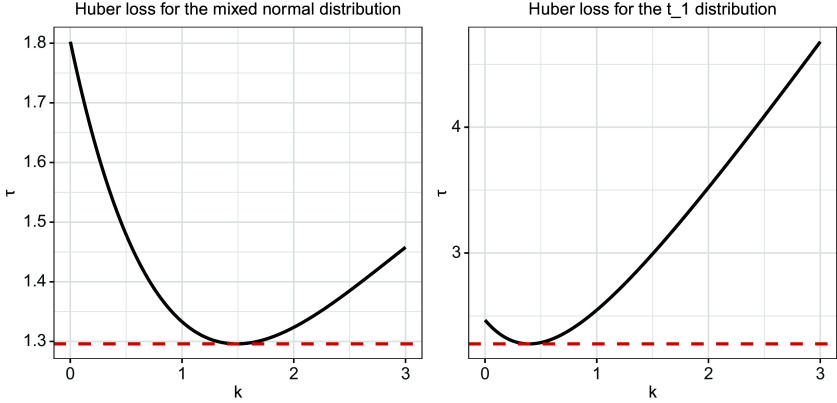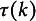 is plotted for 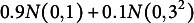 (left) and 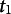 (right). The corresponding red lines are 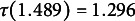 and 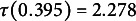 , respectively.

For ease of reference, we also list the optimal 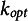 and 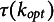 in Table 1 for some error distributions, including the standard normal distribution 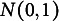 , the Laplace distribution Laplace 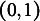 , the mixed normal distribution 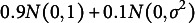 with 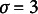 or 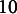 , and the *t* distribution with 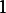 or 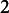 degrees of freedom. In general, the Huber loss with the optimal tuning parameter *k* is more efficient than the LS and LAD losses, since the less 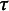 is, the more efficient the loss is. Moreover, to intuitively reflect the variation trend of 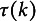 as *k* varies, we also plot the 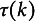 function for a normal mixed and 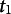 distributions in Figure 2. It is evident that the value of 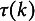 varies dramatically along with the *k* value.

**Table 1:** Optimal *k* and 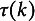 for various error distributions and loss functions

Distribution	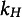	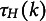	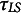	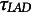
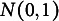	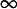	1	1	1.571
Laplace 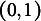	0	1	2	1
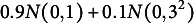	1.489	1.296	1.800	1.803
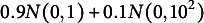	1.222	1.432	10.900	1.897
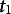	0.395	2.278	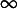	2.467
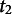	0.692	1.722	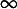	2

### 3.3 Nonparametric selection of Tuning constant

Following (4) and letting 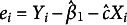 be the residuals, we propose to estimate 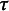 nonparametrically by 
(8)

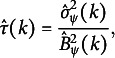

where 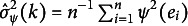 and 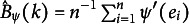 . More specifically for the Huber loss, we have 

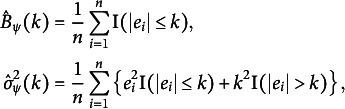

where 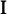 is the 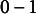 indicator function.

We propose a data-driven procedure that determines the optimal 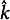 by minimizing 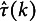 , which is, in fact, similar to Wang et al. (2007) for a linear regression model with a scale parameter 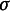 . Our new procedure is summarized in Algorithm 2.

**Figure:**
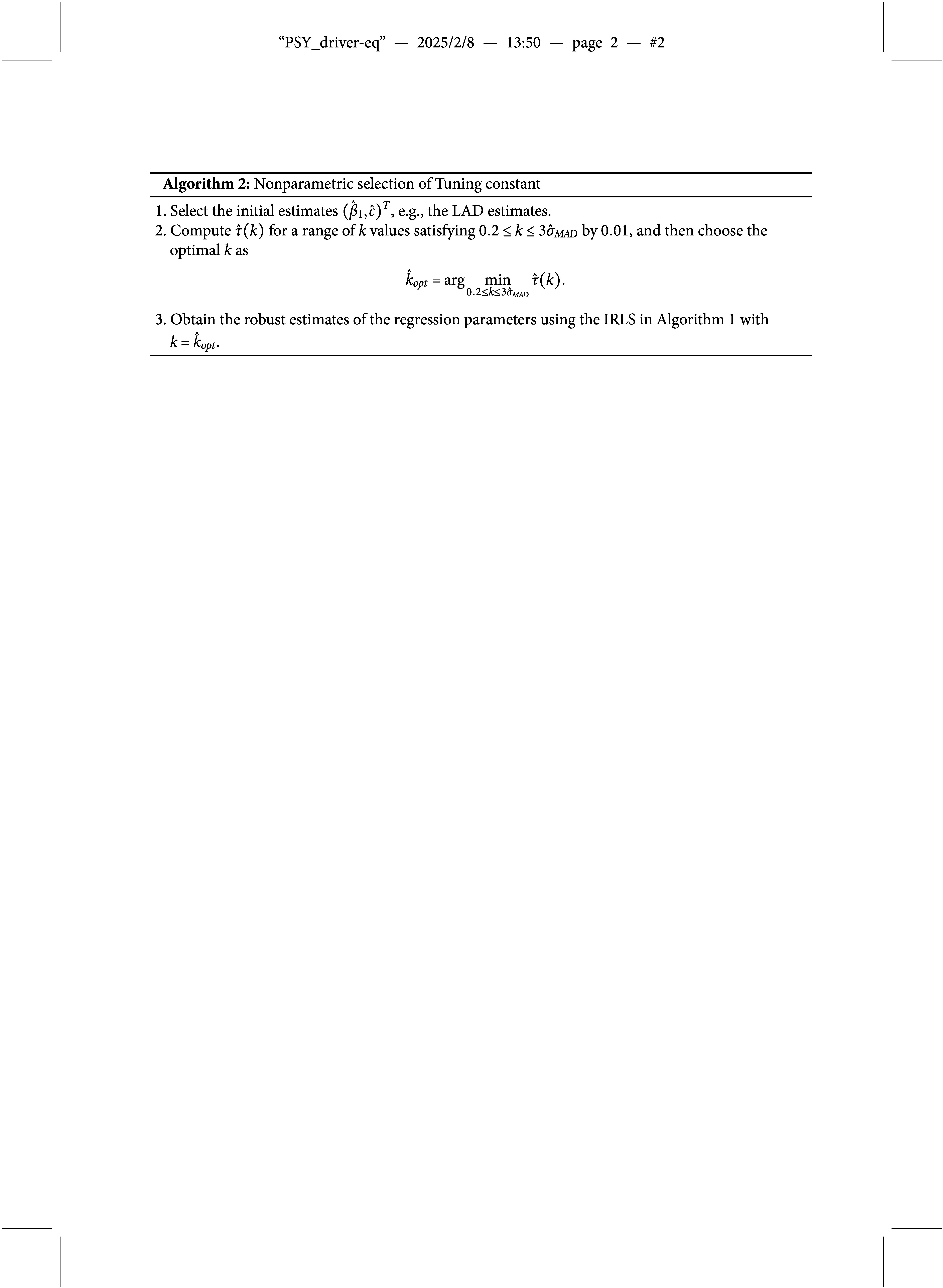

Note that in the algorithm, we have specified the maximum allowable *k* as 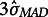 , which is often treated as sufficient since the probability that the errors fall within the interval 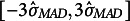 is as large as 99.73% for the normal errors. To further investigate the performance of the proposed method on the selection of tuning constant *k*, we conduct a simulation study and report the results in Table B of the Appendix. When the sample size is large, the selected tuning constant *k* is very close to the theoretical one. Moreover, we note that the standard deviation of the tuning constant decreases dramatically as the sample size increases. These findings coincide with the conclusion in Wang et al. (2007).

## 4 Simulation studies

Two simulation studies are carried out to evaluate the performance of the proposed method. Simulation A compares the efficiency of the three estimators based on the LS, LAD, and Huber losses under various designs, and Simulation B evaluates their type I error rate and power. For the simulation settings, we follow Yuan and MacKinnon (2014) and Wang and Yu (2023) and set 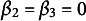 , 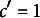 , and 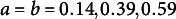 . Moreover, the sample size is set at 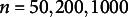 , corresponding to the small, medium and large samples, and four error distributions will be considered including 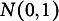 , Laplace 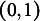 , 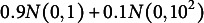 , and 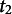 .

For each simulated dataset, we estimate the regression parameters based on the LS, LAD, and Huber losses, and apply the product 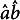 to estimate the indirect effect. Then with 1,000 simulations for each setting, we compute the mean square error (MSE) to assess the estimation accuracy as follows: 

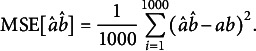



Moreover, we apply the type I error rate and the statistical power to assess the performance of the LS, LAD, and Huber estimators for testing 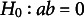 . We use the robust Sobel test (Sobel Z), the percentile bootstrap (PRCT), and the BCa methods to construct the CIs. The type I error rate denotes the probability of incorrectly rejecting the null hypothesis when it is actually true, whereas the statistical power refers to the probability correctly rejecting the null hypothesis when the alternative hypothesis is true. A good testing procedure should control the type I error rate and, meanwhile, it also maximizes the power as much as possible. In practice, the empirical type I error rate (or power) is calculated as the proportion of CIs that do not contain zero when the indirect effect does not exist (or exists).

### 4.1 Efficiency of the LS, LAD, and Huber estimators

The MSE( 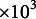 ) and standard deviation (SD 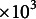 ) of the LS, LAD, and Huber estimators are presented in Table 2 for various designs. Comparing the MSE of the three estimators, we have two main findings. First, the MSE and SD of the three estimators decrease as the sample size increases. Second, the MSE and SD of the Huber estimator are always the smallest or close to the smallest. When the error follows 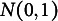 (or Laplace 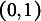 ), the LS (or LAD) estimator provides the optimal estimation. In these two cases, the Huber estimator performs very close to the performance of the optimal estimator. While for 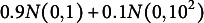 and 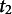 , the MSE of the Huber estimator is the smallest among the three estimators. To conclude, the Huber estimator is more efficient than the LAD estimator when the error distribution is normal, and is more robust than the LS estimator when the error distribution is non-normal.

**Table 2:** MSE ( 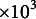 ) and SD ( 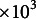 labeled below MSE) for the LS, LAD, and Huber estimators

	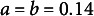			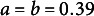			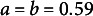		
*n*	LS	LAD	Huber	LS	LAD	Huber	LS	LAD	Huber
	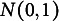								
50	**1.19**	2.22	1.69	**13.9**	22.25	18.2	**6.29**	10.26	8.34
	(2.91)	(4.57)	(3.73)	(10.44)	(15.65)	(13.34)	(21.22)	(31.61)	(27.07)
200	**0.24**	0.39	0.28	**3.83**	5.82	4.42	**1.69**	2.58	1.95
	(0.46)	(0.71)	(0.53)	(2.54)	(3.76)	(2.90)	(5.56)	(8.20)	(6.36)
1,000	**0.04**	0.07	**0.04**	**0.74**	1.14	0.75	**0.32**	0.50	0.33
	(0.06)	(0.11)	(0.06)	(0.45)	(0.76)	(0.47)	(1.04)	(1.73)	(1.08)
	Laplace 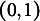								
50	1.34	0.84	**0.82**	6.76	4.92	**4.71**	14.65	11.03	**10.47**
	(3.29)	(1.75)	(1.80)	(11.93)	(7.55)	(7.43)	(23.80)	(16.33)	(15.72)
200	0.24	0.15	**0.14**	1.70	1.08	**1.07**	3.89	2.46	**2.43**
	(0.38)	(0.24)	(0.24)	(2.36)	(1.56)	(1.52)	(5.35)	(3.56)	(3.43)
1,000	0.04	**0.02**	**0.02**	0.34	**0.17**	0.18	0.78	**0.39**	0.42
	(0.07)	(0.03)	(0.03)	(0.49)	(0.24)	(0.26)	(1.14)	(0.55)	(0.59)
	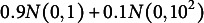								
50	1929.95	21.02	**18.14**	3130.94	73.84	**69.83**	4928.99	152.67	**147.54**
	(4461.83)	(61.24)	(57.06)	(6524.01)	(169.61)	(165.16)	(9212.73)	(315.85)	(308.93)
200	568.45	2.76	**2.53**	1586.29	13.78	**13.42**	3100.87	29.97	**29.39**
	(1386.04)	(9.65)	(8.06)	(3237.90)	(29.33)	(27.12)	(5592.51)	(55.75)	(52.94)
1,000	28.44	0.32	**0.31**	154.49	2.34	**2.29**	340.70	5.33	**5.23**
	(71.51)	(0.53)	(0.49)	(264.92)	(3.34)	(3.23)	(531.80)	(7.54)	(7.36)
	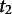								
50	23.61	1.75	**1.52**	75.38	9.82	**8.84**	144.62	21.76	**19.70**
	(379.83)	(3.94)	(3.38)	(931.66)	(15.07)	(13.73)	(1574.78)	(31.82)	(29.63)
200	1.33	0.30	**0.26**	8.73	2.15	**1.90**	19.82	4.89	**4.32**
	(3.64)	(0.53)	(0.41)	(22.65)	(3.07)	(2.61)	(51.53)	(6.80)	(5.89)
1,000	0.65	0.05	**0.04**	3.04	0.36	**0.31**	6.59	0.82	**0.71**
	(9.24)	(0.06)	(0.06)	(14.08)	(0.48)	(0.43)	(26.75)	(1.09)	(0.97)

*Note*: Note that the bold font indicates the samllest MSE among the three estimators under one set of experimental conditions.

### 4.2 Type I error rate and power

We now apply the Sobel Z, PRCT and BCa methods to construct the 95% CI. Note that the medium effect sizes ( 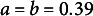 ) will yield a high power even when the sample size is moderate ( 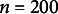 ). Thus to save space, we omit the simulation for the large effect size.

Table 3 report the type I error rates of the three estimators under various designs. When the sample size is large, i.e., 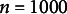 , we note that the type I error rates of the LS, LAD, and Huber estimators are all controlled in most cases. One exception is the LS estimator with the CIs constructed by the BCa method, which was also observed by Fritz et al. (2012) with an explanation that the increased type I error rate is a function of an interaction between the nonzero effect size and the sample size. Another notable situation is that the type I error rate of the Huber loss Sobel test is slightly too high for the mixed normal and 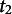 under the small and moderate sample sizes. Possible reasons can be, e.g., the standard error used for the Sobel test 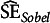 is affected by the Optimizer’s curse (Smith & Winkler, 2006), and/or there is a potential gap between the optimal tuning constant and the one determined by Algorithm 2 in the small sample size. In Appendix E, we have also conducted another simulation study to assess their effect on the standard error used for the Sobel test. The results indicate that the 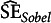 is indeed influenced by the optimizer’s curse, whereas its effect will diminish as the sample size increases. At the same time, the Huber estimator with the fixed 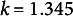 performs better than the Huber estimator with the selected tuning constant (Huber-SEL) in the case of small sample size. Observing this, when the Huber-SEL estimator fails to yield satisfactory results, we suggest to take a moderate tuning constant, i.e., 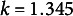 , as an alternative.

**Table 3:** Type I error rates (%) of the LS, LAD and Huber estimators for various designs

		Sobel Z			PRCT			BCa		
	*n*	LS	LAD	Huber	LS	LAD	Huber	LS	LAD	Huber
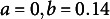	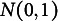									
	50	0.0	0.0	0.3	0.3	0.0	0.2	1.5	2.1	1.4
	200	0.3	0.3	0.5	1.9	0.5	1.0	3.7	3.0	3.4
	1,000	1.4	1.2	1.5	3.1	2.0	2.9	5.7	5.2	5.4
	Laplace 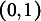									
	50	0.2	0.2	1.5	0.7	0.1	0.3	1.7	2.3	1.8
	200	0.2	0.9	1.6	1.8	1.8	1.7	5.0	4.7	4.4
	1,000	1.9	1.8	2.7	4.1	2.9	3.0	5.9	4.6	4.6
	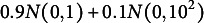									
	50	0.0	1.5	**8.2**	0.9	0.3	0.3	4.7	2.4	2.1
	200	1.4	2.2	5.9	0.2	0.1	0.0	2.5	1.9	1.6
	1,000	2.2	2.5	3.5	1.3	0.7	0.4	3.3	2.5	1.5
	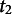									
	50	0.0	0.5	2.9	1.0	0.3	0.6	4.4	3.7	2.2
	200	0.8	0.8	3.8	2.0	1.6	2.1	**7.8**	5.0	4.8
	1,000	2.6	3.6	4.1	4.3	3.4	3.9	6.7	4.6	5.0
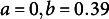	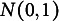									
	50	1.7	0.8	4.2	3.9	1.3	3.0	**7.0**	6.7	6.3
	200	3.2	2.7	4.4	5.2	3.2	4.3	**7.0**	**7.1**	6.2
	1,000	5.3	4.9	5.4	5.5	4.6	5.0	5.4	6.0	5.0
	Laplace 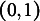									
	50	1.2	1.2	**6.9**	4.4	1.5	3.5	**8.6**	6.7	6.0
	200	3.3	2.6	4.7	5.1	2.6	3.5	**7.4**	5.4	5.0
	1,000	4.5	3.1	5.0	4.7	3.7	4.4	5.3	4.1	4.6
	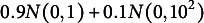									
	50	0.0	0.9	**9.0**	1.4	0.3	0.4	**7.8**	3.3	2.6
	200	5.6	3.7	**7.9**	2.3	0.6	0.9	**8.5**	3.9	4.1
	1,000	3.3	3.4	3.9	4.7	2.5	2.3	**8.0**	5.6	4.8
	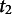									
	50	0.7	1.0	**8.0**	4.2	2.2	3.4	**9.2**	6.8	5.7
	200	2.1	4.8	**8.8**	5.1	4.0	5.6	**10.4**	**7.3**	**7.4**
	1,000	3.9	3.1	5.3	5.6	3.8	4.1	**7.2**	5.4	3.7

*Note*: Note that the bold font indicates the excessive type I error rate which exceeds 6.8% since with 1000 independent simulation runs, the type I error rate of a test with level 0.05 is expected lie in the interval 
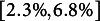
 with probability 0.99, using the normal approximation.

Following the same designs, we report the power of the three estimators in Table 4. For the normal errors, it is evident that the LS estimator not only controls the type I error rate but also achieves the highest power among the three estimators. Nevertheless, for the non-normal errors, the LS estimator is notably lacking in statistical power especially for the mixed normal distribution (e.g., 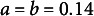 , 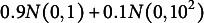 , and 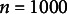 ). In addition, despite that the LAD estimator is the most robust method with respect to the outliers, it however suffers from the efficiency loss and consequently yields a lower power (e.g., 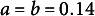 , 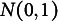 , and 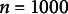 ). In contrast, the Huber estimator makes a trade-off between the efficiency and robustness, in which its power is close to the largest and, meanwhile, it also controls the type I error rate below 5% regardless of the error distribution.

**Table 4:** Power (%) of the LS, LAD and Huber estimators for various designs

		Sobel Z			PRCT			BCa		
	*n*	LS	LAD	Huber	LS	LAD	Huber	LS	LAD	Huber
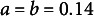	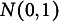									
	50	0.2	0.2	**1.4**	**2.5**	0.6	1.1	**4.9**	4.8	4.3
	200	9.9	4.4	**12.7**	**22.8**	8.3	18.6	**33.5**	17.7	27.8
	1,000	**95.0**	67.4	94.8	**97.4**	80.8	96.9	**98.1**	84.8	**98.1**
	Laplace 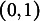									
	50	0.2	0.6	**4.4**	**3.4**	1.7	3.3	8.1	**8.7**	6.4
	200	8.5	22.7	**37.8**	23.7	33.9	**41.1**	34.9	44.6	**51.4**
	1,000	94.0	99.9	**100.0**	96.8	**100.0**	**100.0**	97.5	**99.9**	**99.9**
	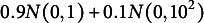									
	50	12.3	5.9	**28.4**	**2.4**	0.8	1.3	**6.1**	3.7	3.4
	200	1.3	15.3	**37.7**	0.8	1.8	**2.5**	3.3	**5.9**	**5.9**
	1,000	3.9	66.5	**89.2**	1.4	16.5	**18.3**	2.7	23.8	**25.1**
	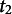									
	50	0.5	0.5	**5.2**	**1.9**	0.7	1.4	**5.3**	4.3	3.8
	200	2.9	12.4	**25.4**	9.1	17.8	**21.7**	15.9	25.4	**29.7**
	1,000	20.3	80.1	**88.5**	36.3	81.2	**87.3**	40.6	83.1	**88.0**
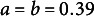	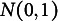									
	50	20.0	7.6	**32.0**	**35.2**	10.9	25.2	**47.8**	23.2	38.7
	200	**100.0**	95.7	**100.0**	**100.0**	98.3	**100.0**	**100.0**	97.5	**100.0**
	1,000	**100.0**	**100.0**	**100.0**	**100.0**	**100.0**	**100.0**	**100.0**	**100.0**	**100.0**
	Laplace 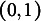									
	50	45.2	47.4	**84.9**	60.2	54.8	**67.1**	68.9	59.7	**75.3**
	200	99.8	**100.0**	**100.0**	99.8	99.9	**100.0**	99.7	99.7	**100.0**
	1,000	**100.0**	**100.0**	**100.0**	**100.0**	**100.0**	**100.0**	**100.0**	**100.0**	**100.0**
	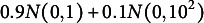									
	50	0.0	24.1	**74.7**	1.8	7.7	**9.6**	6.3	16.3	**18.7**
	200	0.7	73.5	**98.9**	2.5	30.5	**31.3**	8.6	37.6	**36.2**
	1,000	11.8	99.4	**100.0**	9.1	**90.8**	90.2	12.9	**90.9**	90.3
	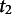									
	50	6.5	17.3	**50.7**	20.0	23.5	**30.1**	27.9	33.1	**36.7**
	200	51.5	95.0	**99.3**	64.1	96.7	**98.6**	66.4	95.4	**98.0**
	1,000	93.3	99.9	**100.0**	93.8	**100.0**	**100.0**	92.9	**100.0**	**100.0**

*Note*: Note that the bold font indicates the maximal empirical power among the three estimators under one set of experimental conditions.

## 5 Real data analysis

In this section, we conduct two real data analyses to illustrate the usefulness of the proposed method. Both the studies show that our newly method can provide a more efficient estimation than the existing competitors for mediation analysis. To promote the practical application, we have also made the R code publicly available on GitHub at https://github.com/pxj66/REMA.git.

### 5.1 Pathways to desistance study

Our first study is to uncover the causal mechanisms between mental health and violent offending among serious adolescent offenders (Kim et al., 2024). In criminology, one possible mechanism is that individuals with mental health issues may be more likely to experience victimization, and this, in turn, may lead to their committing a serious crime. Our data comes from the Pathways to Desistance (PTD) study, which consists of 1354 serious juvenile offenders in two sites, including the Maricopa County in Arizona (N = 654) and Philadephia County in Pennsylvania (N = 700), over the years from 2000 to 2010 (Mulvey et al., 2013). Focusing on the data of baseline interviews, our study contains a total of 1195 respondents after the data cleansing.

Consider the linear mediation model, 

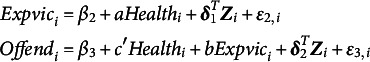

where *Health* (mental health) is the independent variable, *Expvic* (experienced victimization) is the mediating variable, *Offend* (violent effending) is the response variable. In addition, 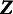 denotes the matrix of other controlled variables including age, gender, enthnicity, family structure, parental warmth, alcohol, marijuana, gang membership, parental hostility, and unsupervised routine activities. We summarize the type and the measure of these variables in Appendix F.

To assess the normality assumption for the errors, we compute the skewness and kurtosis of the residuals of *y* after regressing on *x* and *m* and the residuals of *m* after regressing on *x*, and then report them in Table 5. These values, together with the Kolmogorov–Smirnov (KS) test, clearly suggest a violation of the normality assumption. In view of this, we thus apply our new method to this dataset and also compare it with the existing methods for mediation analysis. Table 6 reports the indirect effects and the 95% CIs constructed by the Sobel Z, PRCT and BCa methods. From the results, we note that the three estimators produce similar and statistically significant indirect effects, whereas the Huber estimator yields the shortest CI.

**Table 5:** Skewness and kurtosis of two regression residuals and the Kolmogorov-Smirnov test for the pathways to desistance study-

	Skewness	Kurtosis	KS test (*p*-value)
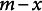	0.3965	2.6953	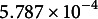
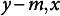	0.9056	4.9128	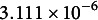
Normal	0	3

**Table 6:** The indirect effect estimates and their 95% CIs based on the LS, LAD and Huber estimators for the pathways to desistance study

		95% CI		
Method	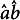	Sobel Z	PRCT	BCa
LS	0.0133	[0.0087, 0.0179]	[0.0087, 0.0188]	[0.0091, 0.0193]
		0.0091	0.0101	0.0102
LAD	0.0113	[0.0067, 0.0160]	[0.0051, 0.0180]	[0.0058, 0.0188]
		0.0092	0.0129	0.0130
Huber	0.0118	[0.0077, 0.0159]	[0.0076, 0.0170]	[0.0076, 0.0171]
		0.0082	0.0094	0.0095

### 5.2 Action planning study

Our second study is to investigate the relationship between action planning and physical activity. In psychology, it is known that the action planning can promote the physical activity, yet the underlying mechanism between them is often unclear. To explore it, an illustrative study has recently been conducted to investigate the action planning promoting the physical activity mediated by the automaticity (Maltagliati et al., 2023), in which a total of 135 participants over 18 years from the tertiary industry were recruited. Participants were asked to wear an accelerometer Actigraph GT3X+, which records their physical activity behaviors and the time of these activities on a notebook for a total of seven days. More specifically in their study, the action planning is the independent variable, measured by four-item Likert scales ranging from 1 (completely disagree) to 6 (full agree). And the automaticity is the mediating variable, measured by four-item of Self-Reported Habit Index ranging from 1 (strongly disagree) to 7 (strongly agree).

Consider the linear mediation model, 
(9)

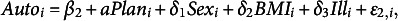



(10)

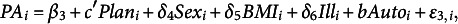

where *Auto*, *Plan*, *Sex*, *BMI*, *Ill*, and *PA* represent the automaticity, action plan of exercise, gender, body mass index, illness, and physical activity of the respondent, respectively.

To assess the normality assumption for the errors, we also compute the skewness and kurtosis of the two residuals, and then report them in Table 7. These values, together with the KS test, suggest a serious violation of the normality assumption for the 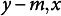 regression residuals. Based on this, we also apply the proposed method to the dataset and then report the result in Table 8. First of all, the three methods produce positive indirect effects from 0.6600 to 0.7594. While for the CIs, only the LAD method shows insignificant outcome in the PRCT CI. At the same time, the Huber loss also yields the shortest CI among the three methods.

**Table 7:** Skewness and kurtosis of two regression residuals and the Kolmogorov–Smirnov test in action planning study

	Skewness	Kurtosis	KS test (*p*-value)
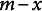	2.6095	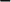 0.1855	0.3582
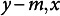	9.7279	2.0349	0.0047
Normal	0	3

**Table 8:** The indirect effect estimates and their 95% CIs based on the LS, LAD, and Huber losses for the action planning study

		95% CI		
Method	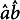	Sobel Z	PRCT	BCa
LS	0.7594	[0.2351, 1.3927]	[0.2714, 1.3755]	[0.3258, 1.4967]
		1.1576	1.1041	1.1709
LAD	0.6619	[0.1796, 1.2521]	[ 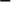 0.1183, 1.3755]	[0.1487, 1.9636]
		1.0725	1.4938	1.8149
Huber	0.6600	[0.4199, 0.9347]	[0.0676, 1.0417]	[0.1470, 1.1820]
		0.5148	0.9741	1.035

## 6 Discussion

This article proposed a novel M-regression for mediation analysis that minimizes the Huber loss function with the optimal tuning constant. The Huber loss can produce a more robust estimator compared to the LS loss when facing outliers and non-normal data, and on the other hand, it can produce a more efficient estimator compared to the LAD loss. Moreover, since the M-estimator may not have an explicit expression for a general loss function, we further proposed an IRLS algorithm for obtaining the numerical solutions. Under some mild conditions on the error distribution, the consistency of the mediation model was also established. Lastly, simulation studies and real data analysis showed that the Huber estimator has a better performance than the LS and LAD estimators.

In the literature, there are two methods commonly used to improve the estimation efficiency. The first method is the M-regression by selecting an optimal loss function from the loss function family. Besides the Huber loss that is among the most commonly used, other popular loss functions include, but not limited to, the Hampel loss (Hampel et al., 1986), the generalized Gauss-weight and linear quadratic losses (Koller & Stahel, 2011), and other general losses (Barron, 2019; Tukey, 1977). When the error distribution is skewed, it is appropriate to adopt the asymmetric Huber and Tukey’s biweight losses for enhancing the estimation efficiency. In the field of microeconomics, the M-regression is done by solving the estimating equations which can be incorporated in the generalized method of moments (GMM), as also introduced in Chapter 6 of Cameron and Trivedi (2005). By making some additional moment conditions, one can obtain more efficient estimators. The second method is to combine the information of quantiles for improving the estimation efficiency, i.e., the composite quantile regression (Zou & Yuan, 2008), the weighted quantile average regression (Zhao & Xiao, 2014), and the combination of difference and robust methods (Wang et al., 2019). Hence as a further direction, it can be of interest to investigate whether the estimation efficiency and power of our new method can be further improved.

## Appendix A Comparing the product and difference estimators

To compare the efficiency of the product and difference estimators, we follow the same simulation design as that for Simulation A in Section 4. Table A shows the MSE (  ) and SD (  ) of the product and difference estimators based on the Huber loss. It is evident that the MSE and SD of the product estimator are smaller than those of the difference estimator. We hence recommend to adopt the product estimator for the subsequent hypothesis testing.

**Table A:** MSE (  ) and SD (  ) for the product and difference estimators based on the Huber loss

			
*n*	MSE	MSE	MSE	MSE	MSE	MSE
	
50	1.52	2.79	7.52	10.21	16.41	20.27
	(3.58)	(6.59)	(12.61)	(17.26)	(25.41)	(30.80)
200	0.27	0.49	1.85	2.45	4.17	4.88
	(0.54)	(1.04)	(2.87)	(3.66)	(6.22)	(6.86)
1,000	0.04	0.05	0.32	0.33	0.74	0.75
	(0.06)	(0.08)	(0.46)	(0.46)	(1.05)	(1.05)
	Laplace					
50	0.81	2.14	4.65	8.44	10.31	16.38
	(1.73)	(4.50)	(7.21)	(13.03)	(15.18)	(25.10)
200	0.14	0.40	1.06	2.06	2.41	4.06
	(0.24)	(0.64)	(1.51)	(2.97)	(3.40)	(5.78)
1,000	0.02	0.07	0.18	0.39	0.41	0.78
	(0.03)	(0.11)	(0.25)	(0.52)	(0.58)	(1.04)
	
50	18.20	22.04	71.08	72.33	150.04	150.36
	(58.27)	(57.54)	(169.64)	(149.64)	(317.91)	(287.81)
200	2.49	3.52	13.45	14.77	29.50	30.84
	(7.72)	(9.05)	(26.57)	(28.12)	(52.20)	(52.54)
1,000	0.31	0.40	2.29	2.56	5.24	5.57
	(0.48)	(0.67)	(3.21)	(3.63)	(7.31)	(7.85)
	
50	1.52	6.53	8.74	20.79	19.41	39.02
	(3.29)	(12.91)	(13.76)	(31.75)	(29.78)	(58.49)
200	0.25	1.31	1.86	5.16	4.25	9.19
	(0.39)	(2.17)	(2.52)	(7.20)	(5.70)	(12.76)
1,000	0.04	0.23	0.31	0.91	0.70	1.59
	(0.06)	(0.34)	(0.43)	(1.31)	(0.97)	(2.33)

## Appendix B An R procedure for selecting of the Tuning constant

From the likelihood perspective, the optimal loss function is given as LS (or LAD) when the error distribution is Normal (or Laplace). Incorporating the relationship of the Huber loss with the LS and LAD losses, the optimal tuning constant is  (or 0) for the Normal (or Laplace) distribution. For other error distributions, the optimal tuning constant minimizes the asymptotic variance of the Huber estimator. More specifically, we can compute  with a sequence of *k*, and then the optimal *k*, which corresponding to the minimum value of  , can be located. In what follows, we provide the R code for two examples, one for the mixed normal distribution  and the other for the  distribution.

**Table B:** The values of Mean, SD and Median for the selected tuning constant

			
*n*	Mean	SD	Median	Mean	SD	Median	Optimal

50	1.001	0.715	0.840	0.980	0.703	0.810	
200	1.539	0.841	1.660	1.529	0.836	1.630	
1,000	2.380	0.509	2.470	2.367	0.515	2.460	
Laplace							
50	0.426	0.296	0.310	0.441	0.303	0.330	
200	0.321	0.163	0.260	0.327	0.165	0.260	0
1,000	0.253	0.075	0.220	0.256	0.075	0.230	

50	0.671	0.466	0.510	0.770	0.590	0.570	
200	0.749	0.447	0.700	0.783	0.477	0.730	1.222
1,000	0.931	0.365	1.005	0.951	0.365	1.030	

50	0.612	0.470	0.430	0.611	0.466	0.430	
200	0.558	0.359	0.440	0.562	0.358	0.450	0.692
1,000	0.558	0.279	0.530	0.551	0.274	0.520	

## Appendix C Selection of the Tuning constant

To evaluate the performance of Algorithm 2, we follow the same simulation design as that for Simulation A in Section 4. Table B presents the Mean, SD, and Median of the selected tuning constant. As the sample size increases, the *k* values are very close to those from the theoretical results. This shows that Algorithm 2 provides a good performance for selecting the tuning constant for practical use. These findings also coincide with the conclusion in Wang et al. (2007). Note that  and  correspond to the chosen tuning constant from Equations (2) and (3), respectively. In practice, when the value of  is small, the value of the efficiency factor  is very unstable. So we set the *k* value ranging from 0.2 to 3  by 0.01.

## Appendix D Asymptotic relative efficiency of the Huber estimator

We first prove that the Huber loss with  produces a 95% efficiency for the normal errors. We focus on Equation (1):  where  . When  , the efficiency factor of the Huber estimator is computed by  , where

with  and  being the cumulative distribution function of the standard normal distribution. Then

and so

This shows that the asymptotic relative efficiency of the Huber estimator relatived to the LS estimator is 95% (Serfling, 2001).

At the same time, using Equation (4.52) on page 84 in Huber and Ronchetti (2009), we have

where  is the probability density function of the standard normal distribution. This implies that, when  , the Huber estimator is resistant to outliers with a breakdown point of  .

## Appendix E Simulation study for Huber loss Sobel test

We conduct a new simulation to investigate the effect of the optimizer’s curse on the standard error used for the Sobel test, which is denoted by  . To achieve this, we consider various tuning constants as alternatives, specifying the number of alternatives as 6, 30, and 291. These numbers correspond to step lengths of 0.5, 0.1, and 0.01, respectively, within the tuning constants’ value range of  . Concentrating on the type I error rates, we set the sample size to be 50, 200, 1,000, or 2,000. We also employ the same true values for the regression parameters and the error distributions as those specified in Section 4. Under 1,000 simulated experiments, we then compute the mean standard error used for the Sobel test (  ) for the Huber loss with the selected tuning constant (Huber-SEL) under the different alternatives, the Huber loss with the fixed tuning constant (Huber-FIX), and the Huber loss with the optimal tuning constant (Huber-OPT).

Table E shows the mean standard error used for the Sobel test (  ) of the Huber-SEL, Huber-FIX, and Huber-OPT estimators under various designs. First of all, the simulation results reveal that the Huber-SEL estimator is indeed affected by the optimizer’s curse, yet this effect diminishes as the sample size increases. For example, let us look at the change in  between different number of alternatives. With  ,  , and  , the  of the Huber-SEL estimator exhibits a gradual decline away from the  of the Huber-OPT estimator, i.e., 31.50, with the values shifting from 27.90 to 27.66, and then to 26.95, as the number of alternatives increases. But when the sample size is 1,000 or 2,000, these values are all close to the optimal value. Secondly, we found that the Huber estimator with the fixed  performs better than the Huber-SEL estimator in the case of small sample sizes. However, as the sample size increases, the Huber-SEL estimator is more close to the Huber-OPT estimator than the Huber-FIX estimator. Combined with the conclusions drawn from Table B, two plausible explanations for the poor performance of the Huber loss Sobel tests in  or  are that there is a potential gap between the optimal tuning constant and the one determined by Algorithm 2 and the  is influenced by the optimizer’s curse. For practical applications, when the Huber-SEL estimator fails to yield satisfactory results, we suggest to take a moderate tuning constant, i.e.,  , as an alternative.

**Table E:** Mean standard error (  ) used for the Sobel test for the Huber-SEL, Huber-FIX and Huber-OPT estimators under various designs

		Huber-SEL			Huber-FIX			Huber-OPT
	*n*							
		
	50	27.90	27.66	26.95	35.09	32.92	31.55	31.50
	200	11.12	10.97	11.10	12.52	11.84	11.27	11.22
	1,000	4.47	4.54	4.56	4.90	4.66	4.58	4.58
	2,000	3.18	3.17	3.16	3.43	3.25	3.19	3.14
		
	50	17.59	16.86	16.62	24.56	26.30	29.24	18.80
	200	7.47	7.30	7.09	9.07	9.58	10.58	7.71
	1,000	3.23	3.18	3.15	3.76	4.01	4.34	3.26
	2,000	2.29	2.27	2.22	2.61	2.79	2.99	2.27
		
	50	19.44	18.70	17.26	26.54	26.52	28.01	25.65
	200	11.13	10.90	10.57	12.18	12.17	13.13	12.12
	1,000	5.32	5.27	5.23	5.45	5.42	5.83	5.40
	2,000	3.80	3.77	3.76	3.85	3.83	4.13	3.82
		
	50	24.22	23.27	23.11	33.95	34.51	40.41	34.45
	200	12.13	11.87	11.33	13.65	14.23	15.57	13.51
	1,000	5.69	5.63	5.55	5.86	6.09	6.60	5.86
	2,000	4.07	4.02	3.98	4.13	4.27	4.62	4.11
		
	50	49.63	49.15	47.46	63.74	61.20	59.88	59.24
	200	27.04	26.62	26.77	30.44	28.93	28.10	27.94
	1,000	12.31	12.34	12.35	13.29	12.70	12.45	12.43
	2,000	8.75	8.73	8.72	9.40	8.95	8.77	8.70
		
	50	36.00	34.75	33.65	49.91	52.23	55.87	39.55
	200	19.45	18.92	18.41	23.25	24.69	26.49	20.03
	1,000	8.87	8.76	8.66	10.23	10.94	11.76	8.96
	2,000	6.33	6.26	6.17	7.20	7.73	8.25	6.30
		
	50	51.66	49.63	46.52	69.66	70.56	74.90	68.72
	200	30.96	30.37	29.44	33.91	33.90	36.57	33.73
	1,000	14.82	14.69	14.58	15.18	15.08	16.25	15.03
	2,000	10.58	10.51	10.48	10.74	10.66	11.50	10.64
		
	50	54.39	52.37	50.59	75.99	78.89	86.58	76.38
	200	32.62	31.70	30.80	36.56	38.11	41.37	36.39
	1,000	15.73	15.56	15.35	16.24	16.83	18.24	16.22
	2,000	11.29	11.16	11.07	11.46	11.88	12.86	11.45

## Appendix F Measures of interesting variables in the PTD study

**Table F:** Measures of interesting variables in the pathways to desistance study

Interesting variable	Data type	Measures
Key variable		
Violent offending	Continuous	The proportion of 11 violent offenses committed during the last 6 months. The example items included beating up somebody badly needing a doctor, being in a fight, and killing someone. The higher the value, the greater variety of offenses the youth engaged in.
Mental health	Continuous	Brief Symptom Inventory consists of 9 subscales. The larger the value, the worse the mental health of the respondents.
Experienced victimization	Continuous	A total 6 items and example questions included “In the past 6 months, have you been chased where you thought you might be seriously hurt?”. The larger value, the more victimizations are experienced.
Control variables		
Age	Continous	14–19.
Ethnicity	Discrete	White, Black, Hispanic, Other.
Gender	0–1	Male = 1, Female = 0
Family structure	Discrete	Single Biological Parent live with the youth, Two Biological Parent with the youth, Other
Gang membership	0–1	The status of the participant’s gang membership for last 6 months.
Parental monitoring	Continuous	Parental Monitoring inventory (9 items) range from “never” to “always”.
Parental warmth	Continuous	Responses ranged from 1 (never) to 4 (always), with higher scores representing more parental warmth.
Parental hostility	Continuous	There are a total of 42 items, 21 items for maternal and paternal, respectively, and responses ranged from 1 (never) to 4 (always), with higher scores indicating greater hostility.
Unsupervised routine activities	Continous	The higher score indicates more unsupervised routine activities.
Alcohol	Continous	The frequency of alcohol drink consumption in the recall period.
Marijuana	Continuous	The frequency of using marijuana in the recall period.
